# Information Theoretic Multi-Target Feature Selection via Output Space Quantization †

**DOI:** 10.3390/e21090855

**Published:** 2019-08-31

**Authors:** Konstantinos Sechidis, Eleftherios Spyromitros-Xioufis, Ioannis Vlahavas

**Affiliations:** 1 Department of Computer Science, Aristotle University, 54124 Thessaloniki, Greece; 2 School of Computer Science, University of Manchester, Manchester M13 9PL, UK; 3 Expedia, 1207 Geneva, Switzerland

**Keywords:** feature selection, mutual information, multi-target, multi-label, clustering

## Abstract

A key challenge in information theoretic feature selection is to estimate mutual information expressions that capture three desirable terms—the relevancy of a feature with the output, the redundancy and the complementarity between groups of features. The challenge becomes more pronounced in multi-target problems, where the output space is multi-dimensional. Our work presents an algorithm that captures these three desirable terms and is suitable for the well-known multi-target prediction settings of multi-label/dimensional classification and multivariate regression. We achieve this by combining two ideas—deriving low-order information theoretic approximations for the input space and using quantization algorithms for deriving low-dimensional approximations of the output space. Under the above framework we derive a novel criterion, *G*roup-JMI-Rand, which captures various high-order target interactions. In an extensive experimental study we showed that our suggested criterion achieves competing performance against various other information theoretic feature selection criteria suggested in the literature.

## 1. Introduction

Many real world applications generate huge amounts of data that create various new challenges, such as learning from high dimensional inputs (features). One way of dealing with big dimensionality is to ignore the irrelevant and redundant features by using a *feature selection* (FS) algorithm [1]. In our work we will focus on information theoretic FS criteria, which quantify the importance of each feature by estimating mutual information terms to capture—the relevancy, the redundancy and the complementarity [2]. Choosing a subset of features that has the highest relevancy with the output space, the minimum redundancy between them and the highest complementarity, helps us to reduce the input space and at the same time keep as much useful information as possible.

At the same time more and more applications need to predict multiple outputs (targets), instead of a single one. Depending on the type of the output variables there are various categories of *multi-target* problems, such as *multi-label classification*, *multi-dimensional classification,* and *multivariate regression*, when the outputs are binary, categorical and continuous, respectively [3]. For example, in computer vision [4], multi-label data are used in automated image annotation, since an image can be associated with a number of semantic concepts. In bioinformatics [5], multi-dimensional learning is used in functional genomics, where a gene or protein is associated with multiple functional labels, since an individual gene or protein usually performs a number of functions. Finally, multivariate regression has been used in ecological modeling in order to predict various target variables that capture the quality of the vegetation [6].

In this paper we focus on deriving novel information theoretic FS methods for multi-target problems. To do so we need to estimate *mutual information* (MI) expressions from finite sample data sets. As the number of selected features grows due to high dimensionality of the input space and as the number of targets is high due to high dimensionality of the output space, the estimated MI expressions become less reliable. To overcome this problem, low-order criteria have been suggested.

Sechidis et al. [7] introduced a framework for generating such low-order FS criteria for multi-target problems by iteratively maximising different composite likelihood expressions, which make various assumptions about the input and output space. By exploring how the different assumptions compare, the authors have found that the best trade-off appears to assume partial independence in the feature and full independence in the target space, a method known as Single-JMI (Joint Mutual Information), details in Section 2. While the partial independence of the feature space has been proven to be useful in deriving FS criteria for single-label data [8], the full independence in the label space ignores the useful information that the possible dependencies between the targets can provide.

Our work, which is an extension of the conference paper in Reference [9], introduces an algorithm that uses the principles of the Single-JMI criterion but at the same time takes into account target dependencies. In the current work, we expanded the preliminary conference paper, by extending the discussion of related work (Section 2), by providing a novel theoretical and sensitivity analysis (Section 3.2 and Section 3.3 respectively), by providing a larger empirical study for multi-label classification (Section 4), including more datasets and competing methods and by providing a novel empirical study on multivariate regression problems (Section 5). The software related to this paper, including implementations of our novel FS criteria, is available at: https://github.com/sechidis.

## 2. Background on Information Theoretic Multi-Target FS

Let us assume that we have a multi-target problem where we observe *N* samples ${\left\{x^{n},y^{n}\right\}}_{n=1}^{N}.$ The feature vector $x=[x_{1}\ldots x_{d}]$ is a realisation of the joint random variable $X=X_{1}\ldots X_{d}$, while the output vector is a realisation of $Y=Y_{1}\cdots Y_{m}$. When the variables of the output space are binary, that is, the alphabet Y is ${\left\{0,1\right\}}^{m}$, the problem is known as multi-label classification, when they are categorical as multi-dimensional classification Y is ${\left\{0,\ldots ,c\right\}}^{m}$, while when they are continuous, that is, Y is $R^{m}$, as multivariate regression [3].

The problem of FS can be phrased as selecting a subset of *K* features $X_{\theta }\subset X,$ where $|X_{\theta }|=K$, that contain as much useful information for our problem as possible. With a slight abuse of notation, in the rest of our work, we interchange the symbol for a set of variables and for their joint random variable. FS methods can be categorized in three groups [10]—filters, wrappers and embedded. Filters are independent of the classifier and they define a scoring criterion (or relevance index) by which they produce a ranking of the features. Wrappers are classifier dependent; they use an evaluation measure to check the performance of the different subsets of features with a particular classifier and they choose the subset with the best performance. Finally, embedded methods are again classifier dependent, since they are part of the learning algorithm and the FS is applied in the training procedure. From the above descriptions we can find the strengths and the weaknesses of each approach. Filters are classifier independent, they are fast and they are less likely to overfit but on the other hand the performance is worse than the classifier specific methods (some of the filters may underfit the data). Embedded methods require some model, which introduces additional assumptions and may be slower than filters but may result to better performance and tend to overfit less than wrappers. Wrappers, because they are classifier dependent, may achieve better performance but on the other hand, they are computationally intensive and tend to overfit more than the other techniques [1,8].

In our work we focus on *filter* methods for FS, which operate under the assumption that the prediction and FS steps are independent [1] or in other words, the selection of features is independent of the classifier or the regressor used. In filter FS, we firstly rank the features according to a score measure and then select the ones with the highest score. The score of each feature should be independent of any classifier and any evaluation measure and it is desirable to increase if the relevancy of the feature with the targets is high, the redundancy with the existing features is low and the complementarity with the existing features is high [8].

### 2.1. Deriving Criteria via Maximum Likelihood Maximization Framework

For single-output problems, that is, the output space is a single variable *Y*, Brown et al. [8] introduced a framework for generating information theoretic FS criteria by phrasing a clearly specified optimisation problem; maximising the conditional likelihood. A greedy forward selection to optimise this objective is: at each step *k* select the feature $X_{k}\in X_{\tilde{\theta }}$ that maximises the following conditional mutual information (CMI):

$$\begin{array}{c} J_{\mathrm{CMI}}\left(X_{k}\right)=I\left(X_{k};Y|X_{\theta }\right), \end{array}$$

where $X_{\theta }$ is the set of the (k−1) features already selected, $X_{\tilde{\theta }}$ the unselected ones and *Y* the single-output target variable. CMI criterion can be written in the following way:

$$\begin{array}{c} J_{\mathrm{CMI}}\left(X_{k}\right)=I\left(X_{k};Y\right)-I\left(X_{k}|X_{\theta }\right)+I\left(X_{k};X_{\theta }|Y\right). \end{array}$$

The first term of the above expression corresponds to the relevancy of a feature with the target, the second to the redundancy of a feature with the set of features already selected and the last term to the complentarity (or conditional redundancy) of the feature with the set of selected features. While the importance of the first two terms is pronounced in the FS literature, the last term has not been traditionally accounted [8]. This term has opposite sign than redundancy, which means that dependent features can be useful, as long the dependence within class is stronger than the overall dependence.

As the number of selected features grows, the dimensionality of $X_{\theta }$ also grows, making the estimates less reliable. To overcome this issue a number of methods have been proposed for deriving low-order criteria. A popular criterion that controls relevancy, redundancy and complementarity, providing a good trade-off between accuracy, stability and flexibility is the joint mutual information (JMI), with scoring function [8]:

$$\begin{array}{cc} J_{\mathrm{JMI}}\left(X_{k}\right) & =\sum_{X_{j}\in X_{\theta }}I\left(X_{j}X_{k};Y\right)\\ \; & \propto I\left(X_{k};Y\right)-\frac{1}{|X_{\theta }|}\sum_{X_{j}\in X_{\theta }}\left(I\left(X_{k};X_{j}\right)-I\left(X_{k};X_{j}|Y\right)\right), \end{array}$$

where the symbol ∝ indicates a ranking equivalent expression for the criterion. The proof for this ranking equivalence can be found in Appendix A.1 of Reference [8]. From the last expression we can see that JMI takes into account all three desirable terms—the score increases when the relevance of a feature is high, when the average redundancy with the features already selected is low and when the average complementarity with the selected features is high.

Sechidis et al. [7] derived two versions of the JMI criterion suitable for multi-output problems, that is, the output space is a joint variable $Y=Y_{1}\cdots Y_{m}$. Their approach was based on the idea of expressing multi-label decomposition methods as composite likelihoods and then showing how FS criteria can be derived by greedily maximising these likelihood expressions. Different decomposition methods lead naturally to different FS criteria. The scoring functions for the two multi-output criteria suggested by Sechidis et al. [7] are the following:

(1) $$\begin{array}{cc} J_{\mathrm{JMI}}^{\mathrm{Joint}}\left(X_{k}\right) & =\sum_{X_{j}\in X_{\theta }}I\left(X_{j}X_{k};Y\right), \end{array}$$

(2) $$\begin{array}{cc} J_{\mathrm{JMI}}^{\mathrm{Sin}\mathrm{gle}}\left(X_{k}\right) & =\sum_{X_{j}\in X_{\theta }}\sum_{Y_{i}\in Y}I\left(X_{j}X_{k};Y_{i}\right). \end{array}$$

The superscripts denote the assumptions over the output space:

Joint-JMI does not make any assumptions and deals with the joint random variable Y. This corresponds to the Label Powerset (LP) transformation in the multi-label literature. The main *limitation* of this method is that Y is high dimensional. For example, in multi-label problems we have up to $\min(N,2^{m})$ distinct labelsets [11], which makes it difficult to estimate MI expressions reliably.
Single-JMI deals with each variable $Y_{i},i=1\cdots m,$ independently of the others. This corresponds to the Binary Relevance (BR) transformation in the multi-label literature. The main *limitation* of this method is that by making the full independence assumption it ignores possible useful information on how the targets interact with each other.

These two versions of the JMI criterion can be seen as the two extreme cases; assuming no independence at all (Joint-JMI) and assuming every outcome it is independent from the rest (Single-JMI).

In a small experimental study, using only two datasets, Sechidis et al. [7] showed that Single-JMI, even though it assumes full independence between the targets, outperforms Joint-JMI, whicht makes no assumptions about the targets. This is happening because the low-dimensional MI expressions in Single-JMI are estimated more reliably from small datasets than the high dimensional MI expressions in Joint-JMI. Next section introduces a novel algorithm that accounts for target dependencies and at the same time keeps the dimensionality of the MI expressions low. Before that we will review other information theoretic criteria suggested in the literature, while a systematic review on multi-label FS methods can found in Reference [12].

### 2.2. Other Information Theoretic Criteria

Yang & Pedersen [13] introduced the first information theoretic multi-label FS method, which ranks the features using the criterion: $J_{\mathrm{MIM}}^{BR}\left(X_{k}\right)=\sum_{Y_{i}\in Y}I\left(X_{k};Y_{i}\right)$. *MIM-BR* ranks the features only on their relevancy with each target independently and it does not take into account possible correlations between features (i.e., redundancy/complementarity). *AMI* [14] is an extension that takes into account redundancy terms but still treats each label independently. *ELA+CHI* [15] uses an Entropy-based Label Assignment, which assigns the labels weights based on label entropy, to transform the label space and then uses the $\chi^{2}$ statistic, a quantity that is asymptotically equivalent to the MI [16], to rank the features. Lee & Kim [17] proposed *PMU*, a criterion that uses the multivariate MI and avoids the computational cost by restricting the number of variables to three. The same authors suggested *FIMF* [18], an algorithm for a computationally efficient information theoretic FS and more recently *SCLS* [19] that introduces a novel way of measuring feature redundancy.

All the above methods were proposed for solving the classification problem (i.e., multi-label) and to the best of our knowledge our work is the first that suggests an information theoretic algorithm that can be used for any kind of multi-target tasks, even on multivariate regression using the default plug-in MI estimator.

At this point we should clarify that in information theoretic FS the scoring criterion, for example, Equations (1) and (2), is combined with a search method which describes how the candidate feature sets are selected. All of the FS algorithms presented so far use greedy forward search, testing each feature in turn for inclusion and adding the one with the highest score. Using a greedy search to present the capabilities of a criterion it is a widely used strategy in the information theoretic FS literature [8]. Apart from the greedy (forward or backward) methods to optimize a scoring criterion, more advanced methods can be used, such as genetic algorithms ([1], Chapter 4). For the remainder of this paper, we will use greedy forward search to test our suggested novel scoring criteria.

## 3. A Novel Framework to Take into Account Target Dependencies

### 3.1. Transforming Output Space via Quantization to Account for Target Dependencies

The main idea behind our approach is to derive a novel representation of the output space $\tilde{Y}={\tilde{Y}}_{1}\cdots {\tilde{Y}}_{m},$ where each variable ${\tilde{Y}}_{i}$ captures the joint information of some group of target variables. After deriving this representation, we will use the following criterion, which we call *Group-JMI*:

(3) $$\begin{array}{cc} J_{\mathrm{JMI}}^{\mathrm{Group}}\left(X_{k}\right) & =\sum_{X_{j}\in X_{\theta }}\sum_{{\tilde{Y}}_{i}\in \tilde{Y}}I\left(X_{j}X_{k};{\tilde{Y}}_{i}\right). \end{array}$$

Group-JMI can be seen as the modification of Single-JMI criterion using ${\tilde{Y}}_{i}$ instead of the initial targets $Y_{i}$. By doing this we keep estimating low dimensional MI expressions but at the same time we take into account target dependencies; each ${\tilde{Y}}_{i}$ captures the information that is shared in a group of target variables. The main challenge is to derive the projected space $\tilde{Y}$ from the initial space Y. Here, we solve this challenge using the following two-step, quantization-based strategy:

- 1st Step—Generate Groups of Target Variables, Using PoT Parameter

In this step we create *m* groups of variables $Z_{1},\ldots ,Z_{m},$ where each group is a random subset of the targets, that is, $Z_{i}\subset Y\;\forall \;i=1,\cdots ,m$. Each group is generated by sampling the set of target variables without replacement and by allowing overlap between the groups. Randomly sampling groups of targets has been extensively used for deriving learning algorithms but not for FS. A famous example is RAKEL  [20], a state of the art method for learning from multi-label data.

Similarly to RAKEL, the number of targets in each group is controlled by a parameter that specifies the Proportion of Targets (PoT) randomly sampled to generate each group. Given, for example, a multi-target problem with m=20 targets and PoT =0.30, 20 groups $Z_{1},\cdots ,Z_{20}$ will be generated, each one consisting of 6(=20×0.30) randomly selected target variables. Assuming binary targets the joint variable in each group may take up to $2^{6}=64$ distinct values, a dimensionality that prevents reliable density estimation unless a very large amount of data is available. To overcome this issue, we introduce a way to derive low-dimensional approximations in the following step.

- 2nd Step—Low-dimensional Approximations via Quantization, Using NoC Parameter

To derive low dimensional representations for each group, we will use the idea of clustering together examples with “similar” output vectors. In the most common case, we assume the Number of Cluster (NoC) is provided a priori. For each group $Z_{i},$ we derive a novel categorical variable ${\tilde{Y}}_{i},$ with the alphabet {1,…,NoC}, that describes the cluster indices of each observation:

$$\begin{array}{c} {\tilde{y}}_{i}^{n}=\mathrm{Clustering}\left(z_{i}^{n},\mathrm{NoC}\right),\forall \;i=1,\cdots ,m,\;n=1,\cdots ,N, \end{array}$$

where the inputs of the clustering algorithm are the target variables of the $Z_{i}$ group and the NoC parameter.

In this work, we use the K-medoids clustering algorithm ([21], Section 14.3.10)—mainly due to its robustness to outliers—but any clustering algorithm that is compatible with the target variables could be used instead. Furthermore, the distance metric can be chosen according to the multi-target problem at hand (e.g., Hamming distance for multi-label classification and Euclidean distance for multivariate regression).

At this point, the problem of estimating the joint (high-dimensional) density of the targets in each group becomes a problem of estimating a discrete distribution of NoC categories. The trade-off is between making no approximations and estimating high-dimensional densities, which leads to poor and unreliable estimates of the MI or deriving lower dimensional approximations through clustering, which leads to more reliable estimates of the MI.

Algorithm 1 provides a greedy forward FS algorithm using our Group-JMI criterion. In Line 6 we need to estimate the JMI between two features, that is, $X_{j}$ and $X_{k}$ and the transformed target variable ${\tilde{Y}}_{j}$ from our sample data. Any MI estimator can be used for this task [22]. In our work we use the plug-in estimator for the MI:

(4) $$\begin{array}{c} \hat{I}\left(X_{j}X_{k};{\tilde{Y}}_{i}\right)=\sum_{x_{j}\in X_{j}}\sum_{x_{k}\in X_{k}}\sum_{{\tilde{y}}_{i}\in {\tilde{Y}}_{i}}\hat{p}\left(x_{j},x_{k},{\tilde{y}}_{i}\right)\ln\frac{\hat{p}\left(x_{j},x_{k},{\tilde{y}}_{i}\right)}{\hat{p}\left(x_{j},x_{k}\right)\hat{p}\left({\tilde{y}}_{i}\right)}, \end{array}$$

where, for example, $\hat{p}\left(x_{j},x_{k},{\tilde{y}}_{i}\right)$ is the maximum likelihood estimate of the joint probability that the random variable $X_{j}$ takes the value $x_{j},$ the random variable $X_{k}$ takes the values $x_{k}$ and the random variable ${\tilde{Y}}_{i}$ takes the values ${\tilde{y}}_{i}$. Estimating these probabilities with categorical features is straightforward, while continuous features can be discretised, for example equal-width discretisation is used often in the FS literature [8,17].

**Algorithm 1** Forward FS with our Group-JMI criterion**Input:** Dataset ${\left\{x^{n},y^{n}\right\}}_{n=1}^{N}$, parameters PoT and NoC and the number of features to be selected *K*.**Output:** List of top-*K* features **X***θ*1: $X_{\tilde{\theta }}=X$▹ Set of candidate features2: Set $X_{\theta }$ to empty list▹ List of selected features3: **for**
i:=1 to *m*▹ Output transformation (where *m* is the number of target variables)4:   Use PoT to generate a random subset of targets: $Z_{i}\subset Y$5:   Derive ${\tilde{Y}}_{i},$ from the cluster indices: ${\tilde{Y}}_{i}=\mathrm{Clustering}\left(Z_{i},\mathrm{NoC}\right)$6: **end for**7: **for**
k:=1 to *K*
**do**8:   Let $X_{k}^{*}\in X_{\tilde{\theta }}$ maximise:9:     $J_{\mathrm{JMI}}^{\mathrm{Group}}\left(X_{k}\right)=\sum_{X_{j}\in X_{\theta }}\sum_{{\tilde{Y}}_{i}\in \tilde{Y}}I\left(X_{j}X_{k};{\tilde{Y}}_{i}\right)$▹ Our scoring criterion10:   $X_{\theta }\left(k\right)=X_{k}^{*}$▹ Add feature $X_{k}^{*}$ to the list11:   $X_{\tilde{\theta }}=X_{\tilde{\theta }}\setminus X_{k}^{*}$▹ Remove feature $X_{k}^{*}$ from the candidate set12: **end for**


### 3.2. Theoretical Analysis

Now we will show that our suggested criterion, Group-JMI, captures all three desirable characteristics of an information theoretic FS criterion—relevancy, redundancy and complementarity. Let us start from Equation (3):

(5) $$\begin{array}{cc} J_{\mathrm{JMI}}^{\mathrm{Group}}\left(X_{k}\right) & =\sum_{X_{j}\in X_{\theta }}\sum_{{\tilde{Y}}_{i}\in \tilde{Y}}I\left(X_{j}X_{k};{\tilde{Y}}_{i}\right). \end{array}$$

Using the chain rule for mutual information, I(AB;C)=I(A;C)+I(B;C|A), the criterion can be written as follows:

(6) $$\begin{array}{cc} J_{\mathrm{JMI}}^{\mathrm{Group}}\left(X_{k}\right) & =\sum_{X_{j}\in X_{\theta }}\sum_{{\tilde{Y}}_{i}\in \tilde{Y}}\left(I\left(X_{j};{\tilde{Y}}_{i}\right)+I\left(X_{k};{\tilde{Y}}_{i}|X_{j}\right)\right). \end{array}$$

The term $\sum_{X_{j}\in X_{\theta }}\sum_{{\tilde{Y}}_{i}\in \tilde{Y}}I\left(X_{j};{\tilde{Y}}_{i}\right)$ in the above is constant with respect to the $X_{k}$ argument that we are interested in, so can be omitted and the criterion gets the following ranking equivalent form:

(7) $$\begin{array}{cc} J_{\mathrm{JMI}}^{\mathrm{Group}}\left(X_{k}\right) & \propto \sum_{X_{j}\in X_{\theta }}\sum_{{\tilde{Y}}_{i}\in \tilde{Y}}I\left(X_{k};{\tilde{Y}}_{i}|X_{j}\right). \end{array}$$

By using the information theoretic identity I(A;B|C)=I(A;B)−I(A;C)+I(A;C|B), the criterion can be written as follows:

(8) $$\begin{array}{cc} J_{\mathrm{JMI}}^{\mathrm{Group}}\left(X_{k}\right) & \propto \sum_{X_{j}\in X_{\theta }}\sum_{{\tilde{Y}}_{i}\in \tilde{Y}}\left(I\left(X_{k};{\tilde{Y}}_{i}\right)-I\left(X_{k};X_{j}\right)+I\left(X_{k};X_{j}|{\tilde{Y}}_{i}\right)\right).\\ J_{\mathrm{JMI}}^{\mathrm{Group}}\left(X_{k}\right) & \propto |X_{\theta }|\sum_{{\tilde{Y}}_{i}\in \tilde{Y}}I\left(X_{k};{\tilde{Y}}_{i}\right)-\sum_{X_{j}\in X_{\theta }}\sum_{{\tilde{Y}}_{i}\in \tilde{Y}}\left(I\left(X_{k};X_{j}\right)-I\left(X_{k};X_{j}|{\tilde{Y}}_{i}\right)\right)\\ J_{\mathrm{JMI}}^{\mathrm{Group}}\left(X_{k}\right) & \propto \sum_{{\tilde{Y}}_{i}\in \tilde{Y}}I\left(X_{k};{\tilde{Y}}_{i}\right)-\frac{1}{|X_{\theta }|}\sum_{X_{j}\in X_{\theta }}\sum_{{\tilde{Y}}_{i}\in \tilde{Y}}\left(I\left(X_{k};X_{j}\right)-I\left(X_{k};X_{j}|{\tilde{Y}}_{i}\right)\right) \end{array}$$

Interestingly, by the decomposition of Equation (8), the first term of *rhs* captures the relevancy of the feature $X_{k}$ and each transformed target variable ${\tilde{Y}}_{j},$ the second term the average redundancy between the feature $X_{k}$ and the already selected features $X_{j}\in X_{\theta },$ while the final term captures the average complementarity between the feature $X_{k}$ and the already selected features, given each transformed target variable ${\tilde{Y}}_{j}.$ The first and the third have positive contribution, while the second negative.

### 3.3. Sensitivity Analysis

This section presents the sensitivity of the proposed algorithm, with respect to the PoT and NoC parameters. We will focus on three multi-label datasets (*image*, *medical*, *genbase*), using three evaluation measures (hamming loss, ranking loss, macro-average F-measure) and in various numbers of selected features (K=1,⋯,50). More details on the experimental setting will be given in Section 4.

**Figure 1:**
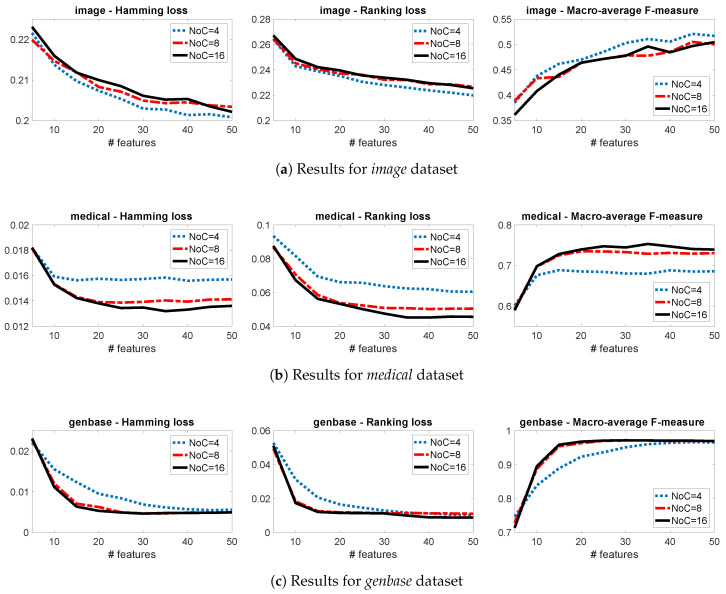
Comparing Group-JMI for various values of NoC with PoT fixed to 0.50.

**Figure 2:**
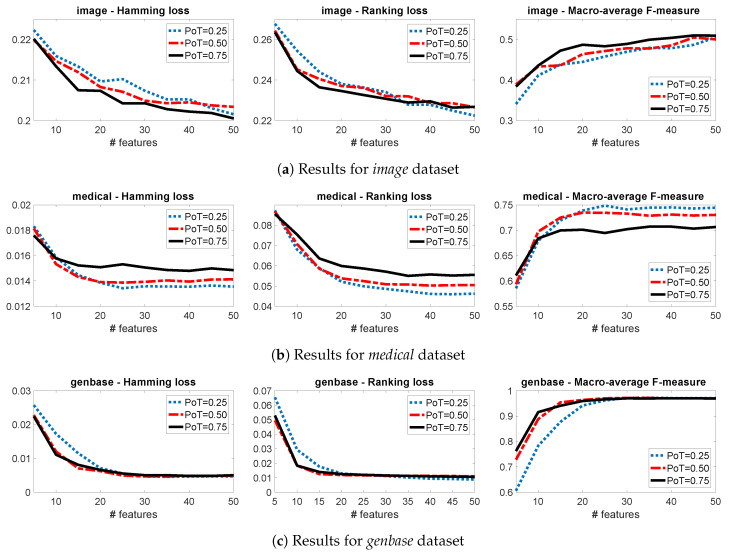
Comparing Group-JMI for various values of PoT with NoC fixed to 8.

Figure 1 shows the performance for different numbers of clusters (NoC) when PoT is fixed to 0.50. We notice that the optimal number is 4 for *image* (Figure 1a), 16 for *medical* (Figure 1b), while for *genbase* there is no clear winner between 8 and 16 (Figure 1c). Figure 2 shows the performance for different proportions of targets when NoC is fixed to 8. We notice that the best performance is achieved by groups that contain 75% of the targets in *image* (Figure 2a), by groups that contain 25% of the targets in *medical* (Figure 2b), while for *genbase* there is no clear winner between 50% and 75% (Figure 2c).

These results highlight the power of our novel parametrisation and the fact that the optimal parameters depend on the intrinsic characteristics of each dataset. For example, the *image* dataset has few labels and distinct label combinations, as a result NoC = 4 is a good approximation, which is not the case for *medical*, a dataset with many labels. On the other hand, the larger the number of labels, the smaller the best PoT. For example in the *medical* dataset, using a PoT = 0.25 means that in each combination we have ∼11 labels, which is already much higher than the total labels of *image* (5 labels). As a result, in *image* we achieve better performance with high values of PoT, while in *medical* with lower.

### 3.4. A Group-JMI Criterion That Captures Various High-Order Target Interactions

One approach to estimate the optimal parameters is by using grid-search on a hold-out set to optimize a specific evaluation measure. However, this approach assumes that a specific multi-target classification/regression algorithm will be used. Unfortunately, this conflicts with the filter assumption—select features independently from the classification/regression algorithm (more details in Section 2).

To overcome this issue, we suggest Group-JMI-Rand, which chooses the parameters for generating each ${\tilde{Y}}_{i},$ uniformly at random from the following pre-specified set:

**Group-JMI-Rand:** PoT chosen randomly from [0.25–0.75] and NoC from {4,...,16}.

By this parametrisation Group-JMI-Rand uses a large number of targets, since to generate each group we sample at random 25–75% of the targets. At the same time clustering keeps the dimensionality of the estimated densities low. To achieve this we are randomly choosing in each group the number of clusters to be between 4–16. In the next section we will show that the above criterion achieves state-of-the-art performance in various datasets and evaluation measures.

## 4. Experiments with Multi-Label Data

We focus on various multi-label datasets with diverse characteristics, shown in Table 1 [23].

To compare the performance of the different FS methods, we train a multi-label classifier using the selected features and evaluate its performance on the testing data using four measures—hamming loss, ranking loss, normalised coverage and macro-average F-measure [11]. Following the FS literature [8], we used a nearest neighbour classifier, which makes as few assumptions as possible about the data and we avoid the need for parameter tuning. For our work we used the multi-label nearest neighbour classifier introduced by Zhang and Zhou [24] and, following their recommendation we set the number of neighbours to 7. We conducted a holdout balanced cross-validation for each experiment—50% of the examples in a given dataset were randomly chosen as the training set for multi-label FS and classifier training and the remaining 50% were used as the test set to obtain the multi-label classification performance to be reported. Each experiment was repeated 30 times and the average testing performance was reported.

To take into account the performance over various values of selected features, we select top-*K* with K=1,⋯,50. For each *K* the method with the best performance (i.e., lowest loss) is assigned ranking score 1, the second best 2 and so forth, and at the end we average the scores across all *K*. This score provides an indication on how well each method performs across a range of *K* values. Finally, for estimating MI the default plug-in estimator was used, while continuous features were discretised into 5 bins, using an equal-width strategy [8].

### 4.1. Comparing Group-JMI-Rand with Other JMI Criteria

Firstly, we will compare our novel JMI criterion, Group-JMI-Rand, with the two multi-label JMI criteria that have been suggested in Reference [7]—Single-JMI and Joint JMI (more details in Section 2). Table 2 presents the ranking score of each FS method averaged across all possible FS sizes (top-K=1,⋯,50). Overall, we see that our method achieves the best performance in 20 out of 36 settings, while Joint-JMI in 13 and Single-JMI in 3. Each setting is a combination of an evaluation loss measure and a particular dataset.

From this set of experiments we can conclude that our initial idea, to derive a criterion that is a trade-off between the two extremes, Single-JMI (assumes independent targets, thus needs to estimate low-dimensional probability distributions) versus Joint-JMI (no assumption at all, thus needs to estimate high-dimensional probability distributions) outperforms both of them. This is happening because Group-JMI-Rand, by using the parameter PoT, randomly groups the labels and as a result it does not assume full independence between the labels. At the same time, by using a quantization algorithm the probability distribution is compressed in a low density specified by the NoC parameter. Interestingly, even choosen PoT and NoC at random from a large pre-specified set of values outperforms the competing methods.

### 4.2. Comparing Group-JMI-Rand with State-of-the-Art Information Theoretic FS Criteria

To test the efficiency of the proposed criterion Group-JMI-Rand, we will compare its performance against six information theoretic FS suggested in the literature—MIM-BR [13], ELA-CHI [15], AMI [14], PMU [17], FIMF [18] and SCLS [19] (arranged in chronological order). More details on the competing methods can be found in Section 2.

**Figure 3:**
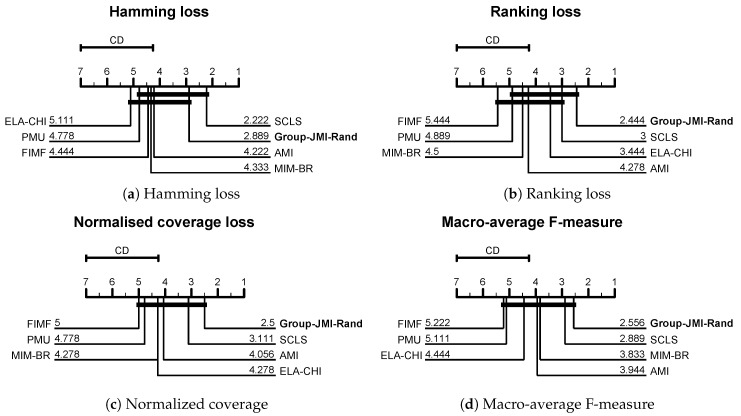
Comparing our suggested FS criterion, Group-JMI-Rand, with state-of-the-art approaches across four evaluation measures: (**a**) Hamming loss, (**b**) Ranking loss, (**c**) Normalized coverage and (**d**) Macro-average F-measure.

In the literature on data mining and machine learning there are various ways on performing statistically sound comparisons between different methods [25,26,27]. In our work we will use the critical difference diagrams (CD), introduced by Demšar [25] and Figure 3 presents our results. For all the CD diagrams of this work, groups of methods that are not significantly different at level α = 0.05 (using the Nemenyi post-hoc test) are connected. The method that achieves the best performance is given a rank of 1, the second best a rank of 2 and so forth.

Our suggested criterion, Group-JMI-Rand, performs better than the competitors in three evaluation measures—ranking loss (Figure 3b), normalized coverage (Figure 3c) and Macro-average F-measure (Figure 3d), while for hamming loss (Figure 3a), a measure that does not take into account label dependencies, the SCLS [19] method performs better. Another interesting conclusion is that our method and SCLS are always in the top-2 positions and in all four evaluation measures there is no statistically significant difference between them. Due to the quantization of the output space, Group-JMI-Rand is more flexible and apart from multi-label data it can be also used to multi-variate regression problems and the next section focuses on this type of data.

## 5. Experiments with Multivariate Regression Data

In this section we focus on various multi-variate regression datasets, shown in Table 3 [28].

As we already mentioned in Section 2, there are no information theoretic FS criteria tailored to multivariate regression data suggested in the literature. For that reason we compare the performance of our proposed algorithm Group-JMI-Rand (using the Euclidean distance for clustering, since we have continuous variables this time instead of binary), against a popular filter FS method, tailored to regression problems—*RReliefF* (Regressional ReliefF) [29]. RReliefF is a nearest neighbor-based feature weighting method for univariate regression problems. In a multivariate regression context, we apply RReliefF separately for each target to get an importance weight per feature and target and then rank the features based on their average importance weight across all targets. We compare the performance of two different variations of RReliefF, *RReliefF 10* and *RReliefF 50* setting the number of neighbours to 10 and 50 respectively.

To compare the performance of the different FS methods, making as few assumptions as possible, we used again a nearest neighbors regression model and predict each target independently. In this set of experiments we set the number of neighbours to be 10, same number of neighbours as in *RReliefF 10*. Finally, the evalutation measure we used is the average Relative Root Mean Squared Error (RRMSE) across all targets, a measure widely used in the multi-target regression literature [28].

**Figure 4:**
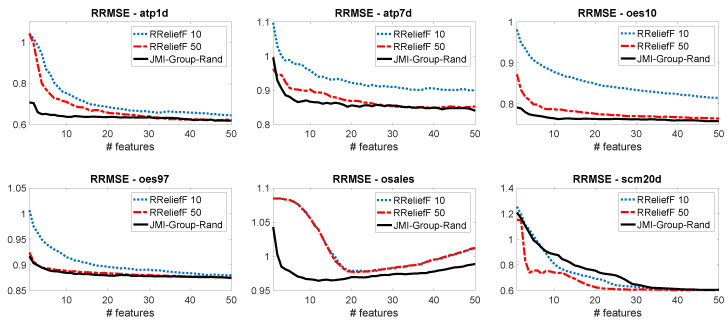
Multi-variate regression experiments. Comparing our suggested criterion with two different versions of RReliefF.

Figure 4 shows that our proposed method *JMI-Group-Rand* achieves the best performance in four out of six datasets (*atp1d atp7d*, *oes10*, *osales*). In *oes97* it achieves the same performance as RReliefF 50, while in *scm20d* the RReliefF methods outperform our information theoretic criterion.

## 6. Conclusions

In this work we presented a FS algorithm suitable for multi-target problems, such as multi-label classification and multivariate regression. Our criterion, Group-JMI, uses the JMI principle to derive low-order approximations of the input space and it clusters similar targets to derive low-order approximations of the output space that capture target correlations. Group-JMI has two parameters—the PoT that controls the number of targets that interact in each group and the NoC that controls the dimensionality of the density that we try to estimate. Under our framework, we suggest the Group-JMI-Rand criterion, which chooses these two parameters at random from a prespecified set of values. On an extensive empirical study across 15 real-world datasets, 10 competing methods and 5 evaluation measures, our proposed criterion Group-JMI-Rand achieves a competitive performance against various other information theoretic FS criteria.

Our future work will focus on providing methods for optimising these parameters. One approach is to use a validation set and minimise a loss of a particular classifier but this violates the filter assumption—selecting the features independently of any classifier or evaluation measure. To overcome this issue our current line of work splits in two directions. For PoT we explore ways of automatically grouping the targets that share some minimum amount of information measured by multi-variate MI. For optimising NoC we explore ways to determine the maximum number of clusters we can have to estimate reliably MI from the available data. This can be done by performing sample size determination for observing given MI quantities with a particular statistical power [30]. Finally, by connecting the problem of multi-target FS with the problem of biomarker discovery in clinical trials with multiple endpoints, we can potentially use Group-JMI-Rand for deriving prognostic and predictive biomarkers in multiple endpoint trials [31]. 

## Figures and Tables

**Table 1:** Characteristics of the multi-label datasets.

Name	Application	Examples	Features	Labels	Distinct Labelsets
*CAL500*	Music	502	68	174	502
*emotions*	Music	593	72	6	27
*enron*	Text	1702	1001	53	752
*genbase*	Biology	662	1186	27	32
*image*	Images	2000	294	5	20
*languagelog*	Text	1460	1004	75	1241
*medical*	Text	978	1449	45	94
*scene*	Images	2407	294	6	15
*yeast*	Bioinformatics	2417	103	14	198

**Table 2:** Comparing the three JMI based criteria in terms of the average ranking score using five evaluation measures: (**a**) hamming loss, (**b**) ranking loss, (**c**) normalised coverage and (**d**) macro-average F-measure. The best method for each combination of evaluation measure and dataset is highlighted in bold.

**(a) Hamming Loss**			
	**Single-JMI**	**Joint-JMI**	**Group-JMI-Rand**
			**(Our Method)**
*CAL500*	2.05	2.10	**1.85**
*emotions*	2.33	1.85	**1.82**
*enron*	2.10	**1.00**	2.90
*genbase*	2.11	**1.71**	2.17
*image*	1.90	2.73	**1.38**
*medical*	2.01	2.86	**1.12**
*scene*	1.80	**1.25**	2.95
*yeast*	1.57	3.00	**1.43**
*languagelog*	1.60	**1.40**	3.00
Total wins	0	4	**5**
**(b) Ranking Loss**			
	**Single-JMI**	**Joint-JMI**	**Group-JMI-Rand**
			**(Our Method)**
*CAL500*	2.20	1.93	**1.88**
*emotions*	**1.57**	2.40	2.02
*enron*	1.75	**1.30**	2.95
*genbase*	2.29	**1.66**	2.05
*image*	1.90	2.77	**1.32**
*medical*	2.11	2.79	**1.10**
*scene*	1.90	**1.15**	2.95
*yeast*	1.52	3.00	**1.48**
*languagelog*	2.62	2.38	**1.00**
Total wins	1	3	**5**
**(c) Normalised Coverage**			
	**Single-JMI**	**Joint-JMI**	**Group-JMI-Rand**
			**(Our Method)**
*CAL500*	**1.75**	2.35	1.90
*emotions*	1.95	2.80	**1.25**
*enron*	1.82	**1.25**	2.92
*genbase*	2.14	**1.44**	2.42
*image*	2.08	2.50	**1.43**
*languagelog*	2.40	2.60	**1.00**
*medical*	1.96	2.84	**1.20**
*scene*	1.57	**1.48**	2.95
*yeast*	1.62	3.00	**1.38**
Total wins	1	3	**5**
**(d) Macro-average F-measure**			
	**Single-JMI**	**Joint-JMI**	**Group-JMI-Rand**
			**(Our Method)**
*CAL500*	1.92	2.25	**1.82**
*emotions*	2.10	2.08	**1.82**
*enron*	2.00	**1.00**	3.00
*genbase*	2.34	**1.49**	2.17
*image*	1.77	2.80	**1.43**
*languagelog*	**1.43**	1.65	2.92
*medical*	2.01	2.84	**1.15**
*scene*	1.77	**1.30**	2.92
*yeast*	1.75	3.00	**1.25**
Total wins	1	3	**5**

**Table 3:** Characteristics of the multi-variate regression datasets.

Name	Application	Examples	Features	Targets
*atp1d*	Airline Ticket Price	337	411	6
*atp7d*	Airline Ticket Price	296	411	6
*oes97*	Occupational Employment Survey	334	263	16
*oes10*	Occupational Employment Survey	403	298	16
*osales*	Online Product Sales	639	413	12
*scm20d*	Supply Chain Management	8966	61	16

## Abbreviations

The following abbreviations are used in this manuscript:

FS	Feature Selection
MI	Mutual Information
CMI	Conditional Mutual Information
JMI	Joint Mutual Information
NoC	Number of Clusters
PoT	Proportion of Targets
